# Recombinase Polymerase Amplification (RPA) of CaMV-35S Promoter and *nos* Terminator for Rapid Detection of Genetically Modified Crops

**DOI:** 10.3390/ijms151018197

**Published:** 2014-10-10

**Authors:** Chao Xu, Liang Li, Wujun Jin, Yusong Wan

**Affiliations:** 1 Biotechnology Research Institute, Chinese Academy of Agricultural Sciences, Beijing 100081, China; E-Mails: xuchao1667@163.com (C.X.); liliang@caas.cn (L.L.); jinwujun@caas.cn (W.J.); 2 Inspection and Testing Center for Environmental Risk Assessment of Genetic Modified Plant-Related Microorganisms (Beijing), Ministry of Agriculture, Beijing 100081, China

**Keywords:** recombinase polymerase amplification (RPA), isothermal amplification, CaMV-35S promoter (P-35S), *nos* terminator (T-*nos*), genetically modified crops (GMCs)

## Abstract

Recombinase polymerase amplification (RPA) is a novel isothermal DNA amplification and detection technology that enables the amplification of DNA within 30 min at a constant temperature of 37–42 °C by simulating *in vivo* DNA recombination. In this study, based on the regulatory sequence of the cauliflower mosaic virus 35S (CaMV-35S) promoter and the *Agrobacterium tumefaciens* nopaline synthase gene (*nos*) terminator, which are widely incorporated in genetically modified (GM) crops, we designed two sets of RPA primers and established a real-time RPA detection method for GM crop screening and detection. This method could reliably detect as few as 100 copies of the target molecule in a sample within 15–25 min. Furthermore, the real-time RPA detection method was successfully used to amplify and detect DNA from samples of four major GM crops (maize, rice, cotton, and soybean). With this novel amplification method, the test time was significantly shortened and the reaction process was simplified; thus, this method represents an effective approach to the rapid detection of GM crops.

## 1. Introduction

The International Service for the Acquisition of Agri-biotech Applications (ISAAA) estimates that millions of farmers cultivated genetically modified (GM) crops over more than 170 million hectares across 27 countries in 2013; the major GM crop species were canola, maize, cotton, and soybean [1]. Due to the constant emergence of new GM crops and their derivatives, consumers are becoming increasingly concerned regarding risks posed by GM crops and products. To protect the consumers’ right to be informed and choose options, many countries have implemented a labeling policy for foods derived from genetically modified organisms (GMOs).

GMO detection is required to implement such an identification system. DNA-based GMO detection methods can be classified as screening, gene-specific, construct-specific, and event-specific detection according to their level of specificity [2]. Screening tests detect exogenous transgenic regulatory elements to determine whether products contain transgenic ingredients. Screening detection is one of the most economical detection methods and also acts as a basis for further GMO identity verification. Although the polymerase chain reaction (PCR) is one of the most widely used amplification methods for GMO screening detection [3], the need for delicate equipment and complicated procedures limit the use of PCR amplification in point-of-use and field settings. Rapid, specific, and highly effective methods for identifying the presence of GMOs in food and feed are important and necessary [4].

Recombinase polymerase amplification (RPA) offers a portable, rapid, and highly specific isothermal alternative to PCR and is ideally suited to point-of-use molecular assays for GMO detection. This technique can be combined with a fluorescent probe for real-time detection, and assays can be completed in a short period of time (within 30 min) at a constant temperature (37–42 °C) by simulating *in vivo* DNA recombination. In the RPA platform, the phage-derived recombinase initially aggregates with the primers and forms nucleoprotein filaments. Then, the filaments scan the template DNA for homologous sequences and catalyze strand exchange at cognate sites [5]. The displaced strand is bound by a single-stranded DNA binding protein, and the primers then are extended by *Bsu* DNA polymerase. Like PCR, this process amplifies DNA exponentially. The DNA repair enzyme exonuclease III is included in the RPA reaction to cleave the probe that is hybridized to the amplicon, thereby separating the fluorophore and the quencher and generating a real-time readout [6]. The use of fluorescent probes provides a convenient method for monitoring amplification events in the RPA reaction. Real-time RPA also has been developed and used for the molecular detection of microorganisms and viruses, such as *Cryptosporidium* [7], *Francisella tularensis* [8], Rift Valley fever virus (RVFV) [9], HIV-1 [10], and other pathogens.

The most frequently used method for detecting GMO material is screening for the CaMV-35S promoter (P-35S) from the cauliflower mosaic virus (CaMV) and the 3' non-translated region of the nopaline synthase gene (T-*nos*) from *Agrobacterium tumefaciens* [11]. In this work, we describe the initial development of a real-time RPA assay to detect P-35S and T-*nos* sequences for purposes of GMO screening and detection.

## 2. Results and Discussion

### 2.1. Primer Design and Screening

RPA primers are typically 30 to 35 nucleotides long. In this study, we tested 24 and 16 primer combinations for the target elements of P-35S and T-*nos*, respectively (Table 1), using 100 copies of GM rice (Kefeng 6 strain) genomic DNA to evaluate the performance of each combination based on a short time to amplification onset (approximately 6–10 min) and ideal plateau fluorescence signal (greater than 500 mV). In the primer screening test, 3 primer combinations successfully amplified the P-35S target, and 2 primer combinations successfully amplified the T-*nos* target. For each target, we selected one primer combination that exhibited good performance (Table 2).

**Table 1:** Primer screening results.

Target Elements	No. of Primer Combinations Tested	No. of Primer Sets Amplified	Fluorescence Signal > 500 mV	Threshold Time of 6–10 min
P-35S	24	3	2	1
T-*nos*	16	2	1	1

**Table 2:** Primers and probes used in P-35S and T-*nos* real-time RPA assays.

Target	Primer/Probe	Sequence (5'–3')	Amplicon (bp)
P-35S	RPA-35S-F	TATCCGGAAACCTCCTCGGATTCCATTGCCCAGC	266
	RPA-35S-R	GTGGGATTGTGCGTCATCCCTTACGTCAGTG	
	RPA-35S-P	TCGTTGAAGATGCCTCTGCCGACAG(FAM-dT)(dSpacer) G(BHQ1-dT)CCCAAAGATGG(phosphate)	
T-*nos*	RPA-*nos*-F	TAAGATTGAATCCTGTTGCCGGTCTTGCGATGA	183
	RPA-*nos*-R	CCTAGTTTGCGCGCTATATTTTGTTTTCTATCG	
	RPA-*nos*-P	CGTTATTTATGAGATGGGTTT(FAM-dT)(dSpacer) A(BHQ1-dT)GATTAGAGTCC(phosphate)	

### 2.2. Sensitivity of the RPA Assays

**Figure 1:**
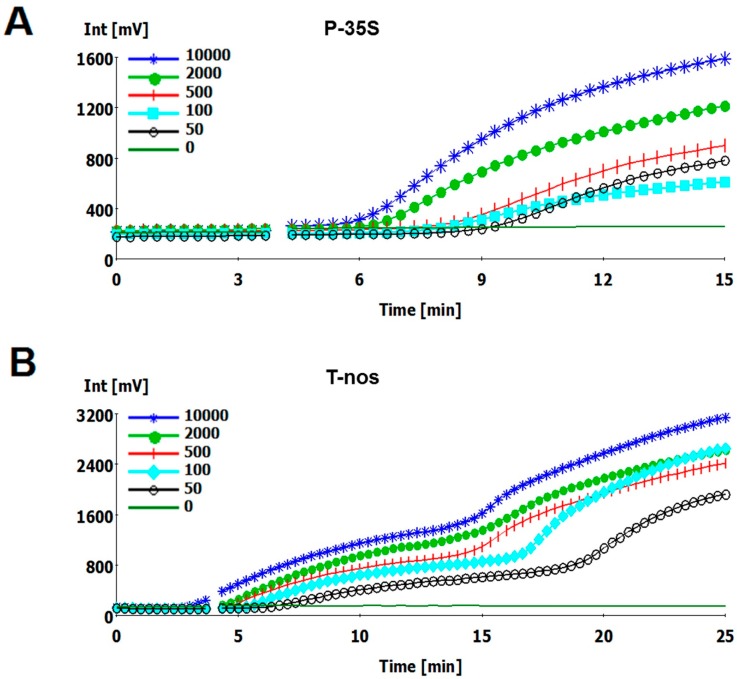
Development of fluorescence intensity (Int) over time for RPA detection for (**A**) P-35S and (**B**) T-*nos*. 10,000, 2000, 500, 100, 50, or 0 copies of genomic DNA were used as the template.

**Figure 2:**
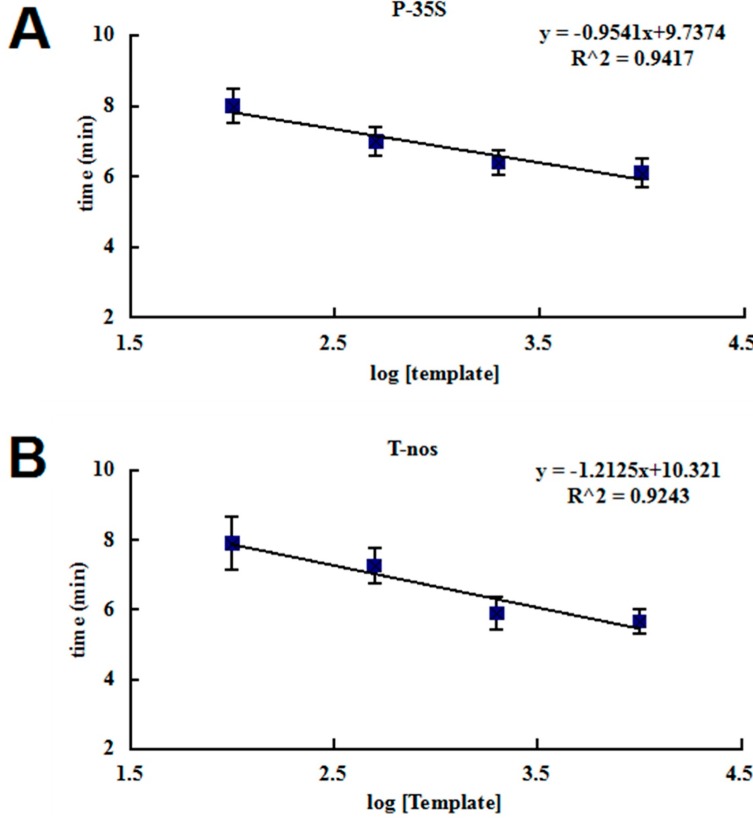
Calibration curves for (**A**) P-35S and (**B**) T-*nos*. Standard curves calculated from the data (shown in Table 3) from 6 runs at 4 concentrations. The *x*-axis represents the logarithm of the estimated copy number of the calibrant, and the *y*-axis represents the threshold time value.

To investigate the sensitivity of the developed RPA assays, 47.5 ng/µL genomic DNA isolated from the GM rice (Kefeng 6) was serially diluted with 47.5 ng/µL non-GM rice DNA. The concentration of positive genomic DNA was 10,000, 2000, 500, 100, and 50 copies/µL at each dilution. In each reaction, 1 µL of diluted DNA solution was used as the template, and 6 replicates per dilution were analyzed. Both of the RPA assays displayed a sensitivity of 100 to 50 detected molecules (Figure 1) and reliably detected 100 copies (Table 3). The probit regression predicted that the P-35S and T-*nos* RPA assays can detect 86.0 and 124.6 copies in 95% of cases, respectively. The mean *R*^2^ coefficient was 0.94 for the P-35S detection method and 0.92 for the T-*nos* detection method (Figure 2), and the slopes of the regression lines were less than the minimum acceptable value of 0.98. Thus, the real-time RPA method is more suitable for qualitative, rather than quantitative detection.

**Table 3:** Sensitivity test for P-35S and T-*nos* real-time RPA assays.

Template Copy Number	P-35S		T-*nos*	
	Threshold Time Values	Positive Reactions/Total Reactions	Threshold Time Values	Positive Reactions/Total Reactions
10,000	6.1	6/6	5.5	6/6
2000	6.4	6/6	6.7	6/6
500	7	6/6	7.3	6/6
100	8	6/6	7.8	6/6
50	ND	5/6	ND	4/6

### 2.3. Application to Practical Sample Analysis

The specificity of the assays was evaluated using genomic DNA from GM maize (Bt11, DAS-59122-7, TC1507, MIR604), GM rice (TT51-1, Kefeng 6, Kemingdao 1), GM cotton (MON15985, MON531), GM soybean A5547-127, and non-GM crops. The DNA concentration of the extracts ranged from 40 to 50 ng/µL. In each RPA reaction, 1 µL genomic DNA was used as the template. Only the positive samples exhibited the existence of P-35S and/or T-*nos* target elements, while no amplification signals were observed for the GM crops without target elements or the non-GM crops (Table 4). The test results are consistent with the AGBIOS GM Crop Database [12] and other references [13,14,15], indicating that two sets of primers can be used as a screening method for rapidly detecting GMOs.

**Table 4:** Results of practical screening test. “+”, theoretically positive; “−”, theoretically negative; P, positive, experimentally verified; N, Negative, experimentally verified.

Species	Event	Regulatory Elements Existing Status		RPA Detection Results	
		P-35S	T-*nos*	P-35S	T-*nos*
maize	Bt11	+	+	P	P
	DAS-59122-7	+	−	P	N
	TC1507	+	−	P	N
	MIR604	−	+	N	P
	Non-GM	−	−	N	N
rice	TT51-1	−	+	N	P
	Kefeng 6	+	+	P	P
	Kemingdao 1	+	+	P	P
	Non-GM	−	−	N	N
cotton	MON15985	+	+	P	P
	MON531	+	+	P	P
	Non-GM	−	−	N	N
soybean	A5547-127	+	−	P	N
	Non-GM	−	−	N	N

### 2.4. Discussion

Over the last decade, nucleic acid isothermal amplification technologies have undergone rapid development [16]. These methods enable nucleic acid testing without temperature-regulating equipment, and several isothermal amplification technologies have been applied to GMO detection, such as nucleic acid sequence-based amplification (NASBA) in combination with microarray detection [17] and loop-mediated isothermal amplification (LAMP) coupled with gel electrophoresis or SYBR Green [4,18,19,20]. Although these novel approaches show high efficiency, NASBA requires template denaturation, and LAMP requires 4 primer pairs to amplify the target at temperatures in the range of 60–65 °C for 60 min; thus, these methods do not satisfy the requirement of simplicity for GMO screening detection.

RPA is another type of nucleic acid isothermal amplification technology. The RPA platform used in this study contains all of the enzymes and reagents necessary to amplify DNA, and only 3 target-specific oligonucleotides are required to conduct a real-time RPA assay. The use of a portable fluorescence detector (Twista, TwistDX, Cambirdge, UK) can reduce the test time to 15 to 25 min. This process is easier and more rapid than other isothermal amplification methods. The Twista detector contains a heated incubation chamber and can test 8 samples simultaneously, and the monitored data can be analyzed by a computer program.

In RPA assays, the amplification reaction can be assessed by gel electrophoresis, fluorescent probes, or lateral-flow strips. Compared with the other two assessment formats, real-time RPA combined with a fluorescent probe is more suitable for rapid and accurate detection. Real-time RPA enables easy visual confirmation of the presence of fluorescence signals, and thus no subsequent operation process is needed. More importantly, the testing process is completed with closed tubes, thereby avoiding product contamination and false-positive test results.

The results of our current study showed that both PRA primer sets of P-35S and T-*nos* could reliably detect 100 copies or more of the targets, a result equivalent to detecting GM content at the level of 0.1%. Although the sensitivity of the developed real-time RPA assays was lower than that of real-time PCR [11,21], it was sufficient to satisfy the requirements of GMO labeling systems in every country [2]. For RPA, primer screening is very important, and the sequences of the oligonucleotides are critical to RPA performance. However, precise rules for obtaining a good primer have not yet been established [22], and several primers may need to be screened to establish a rapid, sensitive RPA detection method for a particular application. As a general rule, when designing optimal primers, regions with high (>70%) GC or AT content, repetitive sequences, and regions with potential secondary structure should be avoided according to the manual produced by the TwistDX company [23]. In summary, we successfully used real-time RPA to detect P-35S and T-*nos* regulatory elements in samples of four major GM crop species. This method that we report generated reliable results for each sample, demonstrating the high specificity of the RPA assay and its suitability for GMO screening.

## 3. Experimental Section

### 3.1. Materials

To prepare samples containing the P-35S and T-*nos* target sequences, GM maize powder (Bt11, DAS-59122-7, TC1507, MIR604), GM cotton powder (MON531, MON15985), and GM soybean powder A5547-127 were provided by the Center of Science and Technology Development, Ministry of Agriculture (Beijing, China). Genuine seeds from GM rice Kefeng 6, Kemingdao 1, TT51-1 and non-transgenic crops were collected by our laboratory.

### 3.2. Extraction of Genomic DNA

Plant genomic DNA from seeds was extracted and purified using a plant genomic DNA extraction kit (TIANGEN Agro-tech Co., Beijing, China) according to the manufacturer’s instructions. The quality and quantity of the DNA samples were measured using a NanoDrop 1000 UV/Vis spectrophotometer (Thermo Scientific, Wilmington, DE, USA) and 1% agarose gel electrophoresis.

### 3.3. Oligonucleotide Primers and Probes

RPA real time fluorescent assays include a forward primer, a reverse primer, and a probe. The primers were designed based on the sequences of P-35S (GenBank accession no. V00141) and T-nos (GenBank no. V00087). Positions 7045–7434 of the P-35S and 1847–2099 of the T-*nos* sequences were selected as target region for primer design and RPA detection. The fluorescent reporter (FAM) and the fluorescent quencher (BHQ1) were conjugated to the T-bases of the probe at internal positions, and an abasic nucleotide analogue such as a tetrahydrofuran (THF) or a 'dSpacer' was located in the central part of the two fluorescent groups, with the phosphate labeled on the 3' end. The RPA primers and probes were synthesized by Sangon (Shanghai, China).

### 3.4. RPA Assays

RPA reactions were performed in a total volume of 50 µL using a TwistAmp Exo kit (TwistDX, Cambridge, UK), 29.5 µL of TwistAmp rehydration buffer, 420 nM each RPA primer, 120 nM RPA probe, 14 mM magnesium acetate, and 1 µL of genomic DNA. All reagents except for the magnesium acetate were prepared in a master mix, which then was added to the freeze-dried reaction tube. Magnesium acetate was added to the tube and spun into the rehydrated material, and the tubes were immediately placed in the Twista tube scanner device (TwistDX, Cambridge, UK) to start the reaction at 39 °C for 15–25 min (for a low template copy number, the strip was removed after 4 min, vortexed, gently spun, and then placed back in the device). Fluorescence measurements were taken every 20 s. For positive samples, the fluorescence signal increased markedly due to successful amplification. The slope validation results (mV/min) are 30 and 24 for P-35S and T-*nos*, respectively. A probit regression was performed using the IBM SPSS for Windows 19.0 (IBM Corp., Armonk, NY, USA) from six replicates results of sensitivity test.

## 4. Conclusions

In this research, we have developed a rapid real-time RPA technique for the detection of P-35S and T-*nos* regulatory elements, which are widely employed in GM crops. This novel method can be easily adapted to other target genes for GMO detection.
